# Considering patient safety in autonomous e-mental health systems – detecting risk situations and referring patients back to human care

**DOI:** 10.1186/s12911-019-0796-x

**Published:** 2019-03-18

**Authors:** Myrthe L. Tielman, Mark A. Neerincx, Claudia Pagliari, Albert Rizzo, Willem-Paul Brinkman

**Affiliations:** 1 Department of Interactive Intelligence, Delft University of Technology, van Mourik Broekmanweg 6, 2628 XE Delft, The Netherlands; 2 TNO Perceptual and Cognitive Systems, Soesterberg, The Netherlands; 3 Edinburgh University, Edinburgh, UK; 4 USC Institute of Creative Technologies, Playa Vista, California USA

**Keywords:** Conversational agents, Risk, Persuasive computing, Chatbots, Assistive technologies, Virtual agents, eHealth, Robotics

## Abstract

**Background:**

Digital health interventions can fill gaps in mental healthcare provision. However, autonomous e-mental health (AEMH) systems also present challenges for effective risk management. To balance autonomy and safety, AEMH systems need to detect risk situations and act on these appropriately. One option is sending automatic alerts to carers, but such ‘auto-referral’ could lead to missed cases or false alerts. Requiring users to actively self-refer offers an alternative, but this can also be risky as it relies on their motivation to do so.

This study set out with two objectives. Firstly, to develop guidelines for risk detection and auto-referral systems. Secondly, to understand how persuasive techniques, mediated by a virtual agent, can facilitate self-referral.

**Methods:**

In a formative phase, interviews with experts, alongside a literature review, were used to develop a *risk detection protocol*. Two referral protocols were developed – one involving *auto-referral*, the other *motivating* users to self-refer. This latter was tested via crowd-sourcing (*n* = 160). Participants were asked to imagine they had sleeping problems with differing severity and user stance on seeking help. They then chatted with a virtual agent, who either directly *facilitated* referral, tried to *persuade* the user, or *accepted* that they did not want help. After the conversation, participants rated their intention to self-refer, to chat with the agent again, and their feeling of being heard by the agent.

**Results:**

Whether the virtual agent *facilitated, persuaded* or *accepted,* influenced all of these measures. Users who were initially negative or doubtful about self-referral could be persuaded. For users who were initially positive about seeking human care, this persuasion did not affect their intentions, indicating that a simply facilitating referral without persuasion was sufficient.

**Conclusion:**

This paper presents a protocol that elucidates the steps and decisions involved in risk detection, something that is relevant for all types of AEMH systems. In the case of self-referral, our study shows that a virtual agent can increase users’ intention to self-refer. Moreover, the strategy of the agent influenced the intentions of the user afterwards. This highlights the importance of a personalised approach to promote the user’s access to appropriate care.

**Electronic supplementary material:**

The online version of this article (10.1186/s12911-019-0796-x) contains supplementary material, which is available to authorized users.

## Background

Advances in technology create opportunities for computer systems to deliver health care interventions and services, either autonomously, or in conjunction with a healthcare professional. As the gap between patient need and workforce availability increases, autonomous digital systems are set to gain a more prominent role. An area in which staff shortages have been particularly acute in many countries is mental health care. While autonomous e-mental health (AEMH) systems offer unique opportunities to support patients, they also come with practical and ethical challenges, chiefly around the outsourcing of patient oversight to computer algorithms.

The World Health Organisation (WHO) states that mental disorders account for 13% of the global burden of disease and hinder economic development on a global level. On a personal level, mental disorders can cause severe problems such as unemployment (with rates up to 90%) and high mortality risks [1]. Yet between 35 and 50% of people in high-income countries with a severe mental disorder receive no treatment. In low-income countries, these numbers rise to 76 and 85%. Several factors contribute to these high numbers, such as the cost of health care, the availability of therapy and the accessibility [2]. Another important issue is the stigma associated with mental ill-health, which often stops people from seeking appropriate support [3]. AEMH’s are interesting, as they might provide a way of breaking down some of these barriers. AEMH’s can be used at home or on a mobile phone, increasing the accessibility of mental health care [4]. The possibility of anonymous care and information might be beneficial for those fearing stigma [5, 6]. Furthermore, AEMH’s offer these advantages for comparatively low cost [4].

AEMH systems offer a means to deliver first-line mental health support autonomously, only triggering full human-based interventions if necessary, thus potentially saving resources for more serious cases [5]. These systems also provide a challenge, however, as human caregivers are minimally involved. This means that AEMH systems themselves need to be able to monitor and assess risks. Moreover, if a situation is detected that the system is not equipped to deal with, it needs to accurately identify risk and be able to activate appropriate human intervention [6]. This fits in with the concept of stepped, or blended care. Current interventions incorporating AEMH systems have a range of ways to deal with situations beyond their scope, depending on the target audience, the technological possibilities and the main purpose of the system.

### Safety in AEMH – Current procedures

Some AEMH systems are designed to encourage vulnerable people to seek professional mental health support. These systems generally employ a combination of questionnaires and free text interaction aimed at assessment. They process users’ textual or physiological inputs and provide them information on their health state [7, 8], as well as informing them of the possibilities of professional human care [9]. As the main goal of these systems is to refer people, they will try to convince people to contact a professional in most situations.

Therapies delivered via AEMH systems (such as cognitive behavioural therapy or exposure therapy) are being developed for several mental health conditions, but specific safety procedures are mainly a feature of those targeting disorders with increased suicide risk. Most current AEMH systems still outsource risk management tasks to a human (e.g. via remote monitoring of symptoms), while the system focuses on automating the delivery of the intervention. An example of a system in which the safety procedure is fully automated is Help4Mood, a home-therapy system for patients with depression [10]. This system is equipped to screen for suicide risk. If a first risk threshold is reached, the system will provide the user with a list of options to deal with the situation, ranging from taking a bath to telephoning a 24/7 suicide help line with human caregivers on the other side. Moreover, if a higher threshold is reached, a human caregiver will step in [11]. Similar work was done with SUMMIT, which focused on reducing depressive episodes [12]. However, in this case a human therapist is involved in monitoring and screening the online forum that is a part of the system. 24/7 help options are made available to users although, in serious cases, a caregiver is automatically alerted. This screening by human caregivers is also applied in the *Reframe IT* intervention to reduce suicide risk [13]. A slightly different approach is taken in a therapy system for post-traumatic stress disorder (PTSD) [14], where patients are given the option to always contact a human caregiver via a message system if they wish, although this is not necessary to follow therapy. Moreover, this same caregiver is able to monitor the questionnaire scores of all patients. Another example of a system for PTSD incorporates weekly phone calls to a therapist as check-up [15]. Even when patients will mainly take medication and not follow therapy, AEMH systems could be applied to facilitate monitoring. For example, the Health Buddy, a monitoring tool for schizophrenic suicidal patients, is a mobile device in which patients answer questions. Answers are monitored by human clinicians who decide if interference is necessary [16, 17].

Patient safety is important for mental health care interventions, and in many AEMH systems there is still a human in the loop who does the risk assessment. These blended-care solutions are very promising, as they can help to decrease demand on professional therapy services while retaining some of the advantages of human care. However, this approach might not always be ideal, or even possible. Key goals of AEMH systems are to reduce the cost and increase the accessibility of therapy. Scalability is crucial to achieve these objectives but having a human in the loop severely limits the scalability of blended interventions. Another important advantage of fully AEMH systems is that they might be used anonymously, lowering the threshold for patients afraid of stigmatization. Web-based applications are promising because they combine this anonymity with being widely accessible [18, 19]. However, a web service that can be used anonymously is limited in incorporating a human caregiver to check for risks. It is therefore important to also have a better understanding on how these AEMH systems themselves should deal with situations beyond their scope. How to determine if a situation requires scaling up to human care, and how to ensure that this scaling up actually occurs.

### Safety protocols

Risk situations occur in all types of mental-health care, and to understand how AEMH systems should deal with them, routine care seems a good starting point. A key difficulty in translating conventional risk management protocols for AEMH systems, is that they are usually formulated as guidelines and thus rely heavily on humans to interpret the content in relation to the context. For this reason, they are often under-specified. An example is the guideline that a caregiver should explicitly ask for suicidality when a person expresses despair [20]. An AEMH system, however, would then first need to correctly identify an expression of despair. In comparison, safety protocols used in research are often more explicit and unambiguous, given the importance of replicability and scientific control.

Study protocols generally identify several different steps in the risk detection process. A first step is information gathering, as identified by Knight et al. [21] and Sands et al. [22] in studies considering practices in risk assessment by phone. Belnap et al. [23] describe a study protocol for suicide screening and incorporate triggers as a first step. In all cases, this first step is meant to identify whether a problem might exist. In phone interviews information gathering is generally described as conversation and open-ended questions, which are less suitable for translation into formal protocols. However, study protocols as described by both Belnap et al. and Rollman et al. [24] identify two possible manners of initial information gathering for suicide detection; namely spontaneous mention of the risk, or a flag on routine screening with scales. After this first step, Belnap et al., Sands at al. and Knight et al. all identify the decision-making stage, or the actual risk assessment. In this stage the professional makes the decision whether a risk actually exists. Sands et al. determine several factors important in making this decision, including self-report measures of behaviour, mood and thought, as well as the duration of the complaint and a person’s history. In the case no professional is included to ask open questions, as in Belnap et al., a specific scale is used to determine risk. If the decision is made that action needs to be taken, the final step is to meet the patient’s needs. This can be to automatically contact a clinician, as advised by Belnap et al., but also to simply refer a patient, as mentioned by Sands et al. It is important to note that when referring a patient, the patient does need to be informed of this plan first and needs to consent. Knight et al. describe recommendations instead of referral, asking the patient to self-refer by contacting a human caregiver. They also note that the caregiver might offer advice for symptom management. As these studies all consider the formalisation of risk detection for mental health, they give valuable insights into what formal protocols for risk assessment might look like for AEMH systems. Dividing risk detection into an information gathering and a decision-making stage, using scales to screen for disorders, and informing patients before automatically referring them, are all aspects that are incorporated into the protocols presented in this paper.

The goal of this paper is twofold. Firstly, it describes protocols on how to detect and act on risk situations in AEMH systems, based on existing safety protocols, the literature and expert input. Secondly, it presents an experiment on the protocol describing how to motivate users to self-refer by adapting the referral strategy to their situation. The following section will first describe the protocols and their underlying theories, and next the methods for the experimental study. Section 3 contains the results of this study, as well as a brief discussion on their meaning for the protocol. The final section of this paper presents an overall discussion of our findings both regarding the protocols and the experimental results.

## Methods

### Risk protocols

#### Methods

Structured interviews were held as starting point to developing theoretical models for safety procedures for AEMH systems. Eight experts took part, each with a scientific background in clinical psychology or human-computer interaction, or with experience as therapist using technology for treatment. The conversations took suicide risk in PTSD therapy as starting point, but also allowed for discussions about other types of risks and systems. The main discussion points that arose from these interviews were presented in a workshop on e-health for virtual agents [25], and were further discussed with the participants. The results of these discussions were combined with previous research on safety protocols for mental health care. This resulted in three new protocols that describe risk detection and subsequent actions by AEMH systems. These models were specifically designed to be applicable to a broad range of mental health problems, risks and systems. As such, the models are general and do not, for instance, cover instruments to measure specific risks. They should therefore be taken as a starting point and need to be operationalized for every specific AEMH they are applied to. This might mean filling in the blanks, but in some cases also adapting the protocol to better fit the solution.

The following section first presents a model for detecting if a situation might warrant the attention of a human caregiver (referred to in the subsequent sections as Model 1). It then describes two approaches for dealing with such risk situations (referred to as Models 2 and 3).

Model 2 describes a protocol wherein the system automatically contacts a caregiver, Model 3 a protocol for motivating the patient to actively seek human care themselves. The latter is especially relevant in systems where not all patient data are known, such as anonymous web services, where auto-referral is not an option. Guided self-referral may be preferable because of such contextual constraints, or where the trigger point for automated risk thresholds is set too low or high, or where an instruction alone may be insufficient to motivate self-referral. In order for the latter approach to be successful, it is important that the system is designed in such a way as to *motivate* the patient to seek help as effectively as possible. The third model describes this process.

#### Resulting protocols

##### Detecting risk (model 1)

**Fig. 1:**
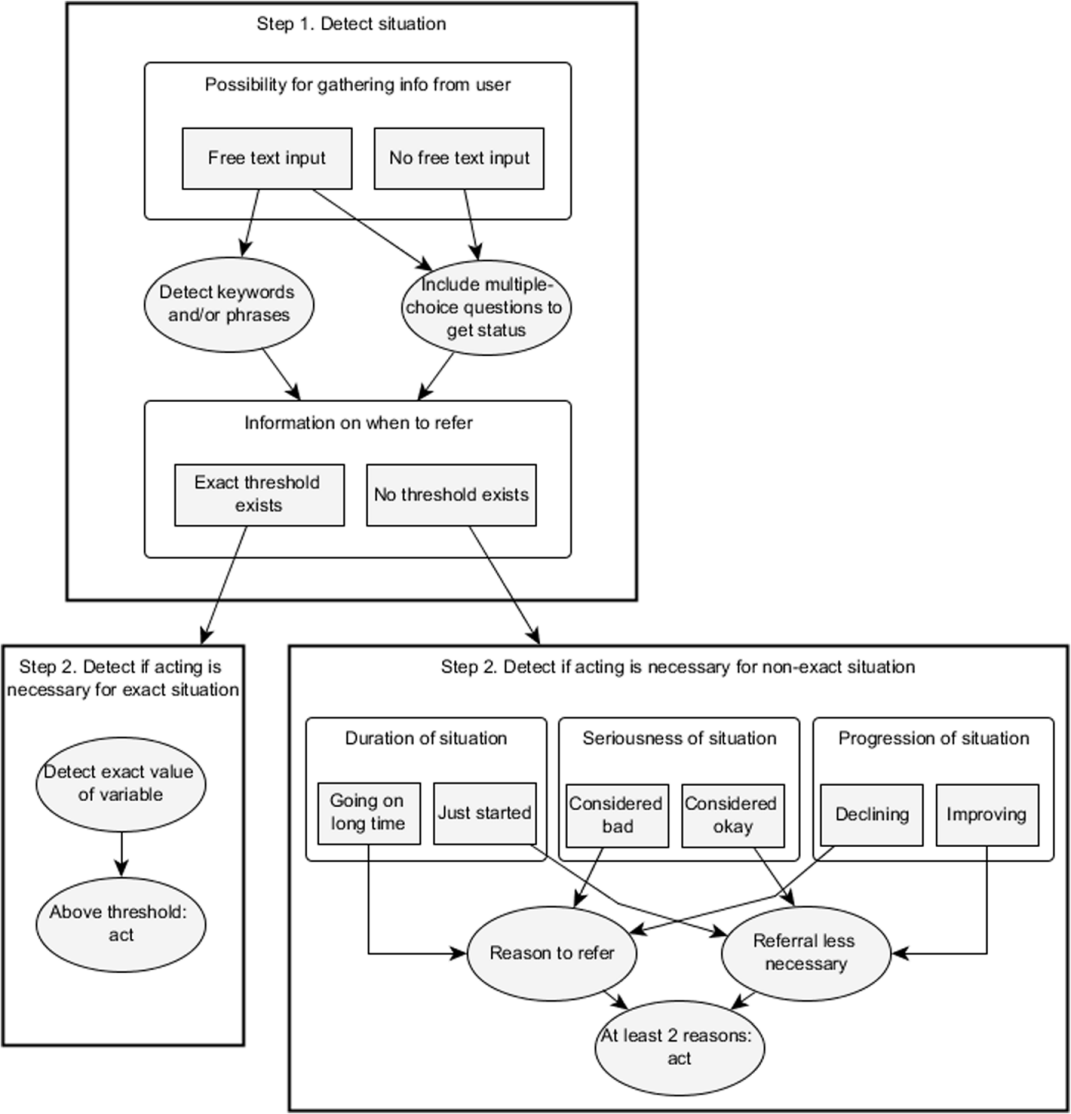
Detection model. Step 1 is to detect if a risk might exist, which can be done by text detection or by including specific questions, depending on the possibilities within the system. Step 2 is to detect if the situation is severe enough to refer to human care. If a threshold value on a questionnaire exists, this can be used for the decision. If not, the duration, severity and progression of the situation could be taken into account to make this decision. Although dependent on exact implementation and situation type, the model gives the guideline of referring if at least two of these values are negative

The *detection model* is presented in Fig. 1. It is important to note that this model does not identify exactly what risk situation is to be detected. Before the detection model can be applied, it is important to establish exactly what risks exist in the user group. The most common example is suicide, which for instance is a risk for PTSD patients [26], but some systems might also wish to detect other risks, such as substance abuse [27]. After this has been established, the detection model identifies two steps:

1. The first is detecting that a possible risk exists, depicted in the top half of the model.
2. The second step is to determine if that risk situation is severe enough to warrant referral, depicted in the bottom half of the model.

Exactly how to detect a risk situation depends on two factors that might differ per AEMH system. The first factor is what type of input the AEMH system gets from the user. Some systems allow free text input from the user [8] and work as a type of chatbot, while others work with closed questions and questionnaires [14]. A system with free text input is capable of screening the input on keywords and phrases related to risk situations. Additionally, it might include specific questions for risk screening. A system without free-text input would need to rely on the second screening method.

To determine how to detect a risk, the second factor is whether there is a measure for the risk situation that can be used to establish a threshold for referral. Questionnaires exist for many situations and their scores may be used to determine when a situation warrants human intervention. For instance, question 9 of the patient-health questionnaire for depression has been shown to be correlated with suicide [28]. The exact threshold score needs to be established in concord with clinical specialists. It might also be necessary to adapt the threshold during use if it becomes clear that too many or too few situations are detected. It is important to note that posing questionnaires to establish risk thresholds might only be appropriate after a possible risk situation has already been detected, which is why this action is not located in the detection part of this model. Especially for suicide, posing unwarranted questions about suicidal ideation might not be the preferred approach. Suicide contagion is a well-studied phenomenon where hearing about suicide prompts suicidal people to commit suicide [29]. This means that care should be taken to only pose suicide questionnaires when the situation already indicates this might be a problem. For example, a suicide prevention phone line might directly start inquiry about suicidal ideation as the fact that a person is calling implies this is relevant. However, a general first-aid phone line would only start posing those questions after further indication to its relevance have been given.

Because questionnaires are not always available, this model also describes a second approach to determining if a risk situation is severe enough for referral. This approach is meant as a guideline of what to take into account during this decision. However, the exact procedure might have to be changed to reflect specific risk factors and situations. For instance, suicide will be a cause for referral quicker than sleeping problems, and when implementing this protocol such factors should be taken into account. This protocol does not claim to be directly applicable to all types of systems and risks, but does offer a starting point in situations where exact scales and questionnaires are not applicable. The model in Fig. 1. considers three factors in determining if a situation warrants referral to human care:

1. The first is duration of the situation, how long has this situation been going on.
2. The second factor is the severity of the situation.
3. The third is the progression, is the situation getting worse or getting better.

As a default, the model recommends that if at least two of these three factors are negative, the user should be referred to human care. Negative in this case is if the situation has been going on for a long time, it is severe, and it is getting worse. Obviously, exactly what warrants as a severe situation, or what is a long duration, should be determined per system and situation. This also holds for situations that are in the middle, such as a situation that neither improves nor gets worse. Depending on the system and risks involved, the number of negative factors necessary for referral can also be expanded or reduced to better reflect the situations that might occur.

##### Auto referral (model 2)

**Fig. 2:**
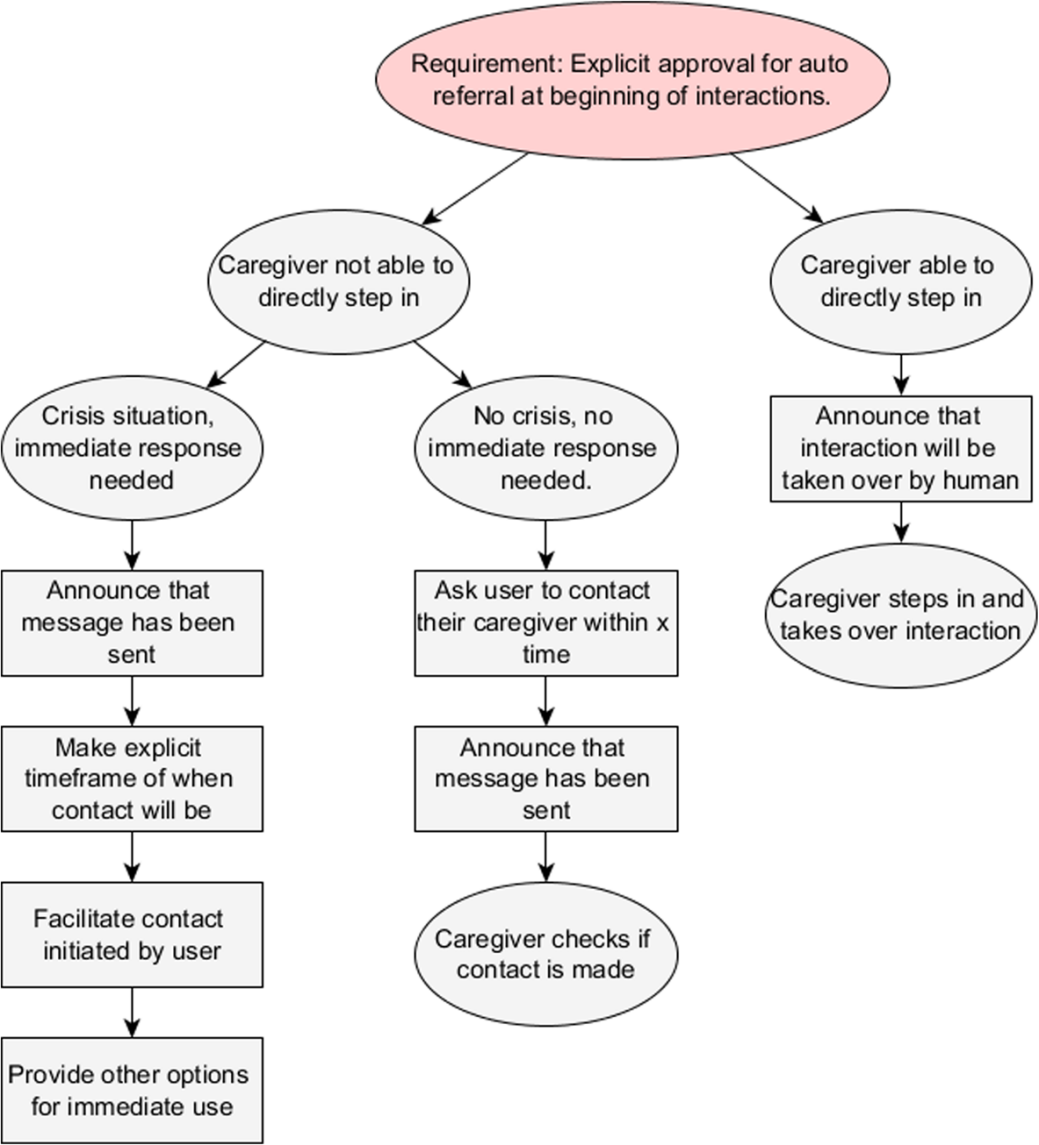
Auto referral model. To automatically refer patients it is important that they have given consent to do so beforehand. If a caregiver is able to directly step in, this will only have to be announced before they take over the interaction. If this is not possible, the model distinguishes between crisis and no crisis situations. In a direct crisis situation, the caregiver is alerted and prompted to act, while the user is also given contact information. If no direct crisis is present, the incentive for seeking contact lies with the user, while the caregiver is still informed as safety net

In some AEMH systems it is possible to automatically refer patients back to a human caregiver. In all cases where automatic referral can take place, it is important that the patient is informed beforehand and has approved this procedure [6]. This ties in with the ethical guidelines to protect the patient’s confidentiality and privacy, and to respect their autonomy [30]. Figure 2. outlines the protocol for an automatic referral.

The recommended actions for automatic referral firstly depend on whether a human caregiver is available to directly step in, for instance by taking over from a chatbot [31]**.** If this is possible, the AEMH system merely needs to inform the patient that they will be interacting with a human, before letting the caregiver take over. Such escalation is not always possible, however, for instance because a caregiver is not always available, or the system does not allow direct intervention. In this case it is important that the users of the AEMH system are known to caregivers before they start [32] [12], as contact details of the user need to be available to the caregiver. In these cases, model 2 distinguishes between direct crisis situations and situations without direct crisis. In the first case, the system directly contacts the human caregiver involved, informs the patient of that fact, and also states when the patient can expect contact. It is important to manage this expectation as caregivers might not always be able to react quickly. Care should be taken that the patient does not believe they are forgotten by their caregiver. Additionally, the system should provide options for the patient to contact the caregiver themselves, as they might be able to respond quicker. This is also relevant in cases of system or network failures. If the patient’s internet network fails it might be impossible for the system to send out a message and the patient should know who to contact and how. Finally, the system should provide some short-term options for the patient to reduce the risk, in case it takes a little while for the caregiver to respond. In case no direct crisis is detected, the model recommends that the patients themselves are asked to contact a human caregiver. Additionally, a message is still sent to the caregiver in case the patient does not comply with the request. Only if the patient does not contact the caregiver within a certain time frame will the care giver seek contact.

##### Motivate to self-refer (model 3)

**Fig. 3:**
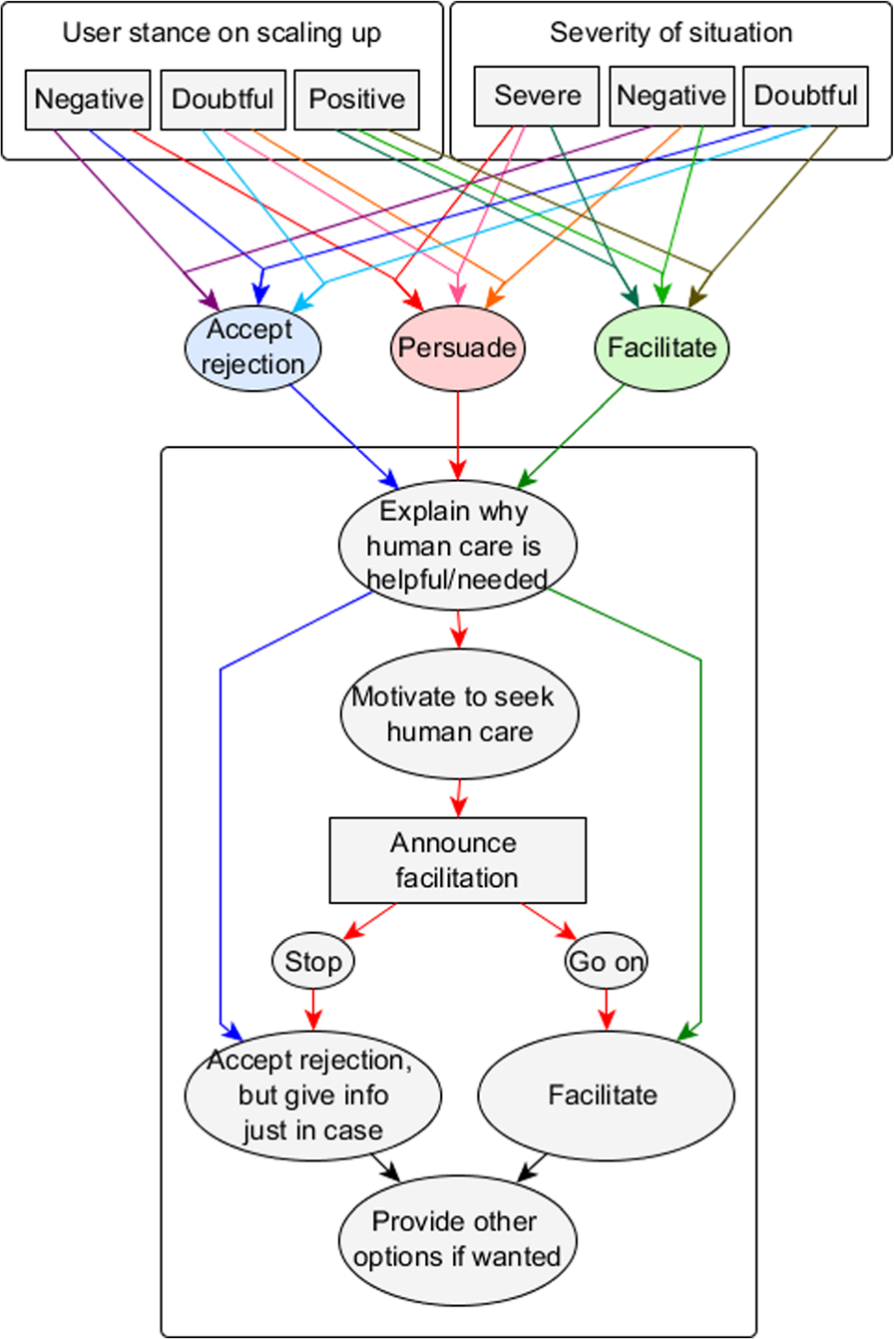
Motivation model. Persuade the patient to self-refer

In some cases, an automatic referral to human care is not preferable, or even possible. Patient anonymity is often an advantage to AEMH systems because of the stigma that still surrounds mental health care [3]. In such anonymous situations the AEMH system does not know anything about the patients, except what they entered into the system themselves. If such a system detects a risk situation, it should generally try to persuade the patients to undertake action themselves and contact a human care giver. Figure 3 presents recommendations on how the system should adapt its strategy for referral to the user’s situation.

The model presented in Fig. 3. has three overall goals, the first is to get as many users as possible to self-refer. The second goal is to give the user the feeling that they are heard and taken seriously by the system. This ties in with the third goal, which is to get users to return to the system if they experience problems again in the future, or if their problems worsen. The protocol proposes that to achieve these goals, the system can personalise its referral strategy to the situation of the user. Three different types of situations are distinguished by how likely users are to self-refer initially, and how important it is that they do so. In other words: how their initial stance is towards seeing a human and how severe their situation is. In every situation, the model proposes a different strategy. Both this division into situation-based strategies and the specific actions within each strategy are based on several theories of behaviour change.

Social judgement theory first identified the concept of latitude of acceptance, which defines the space of options a person finds acceptable [33]. If a suggestion is made in a person’s latitude of acceptance, the user is likely to accept this suggestion. Aside from this latitude of acceptance a user also has a latitude of noncommitment and latitude of rejection. Any suggestion made in the latitude of rejection will likely fail, and additionally make the user even more opposed to the idea. The latitude of noncommitment lies in-between. This paper identifies three situation types which each place the user in one of these three latitudes. The first situation type we will call *accept care,* which is defined by a very positive stance towards seeking human care, placing the user in the latitude of acceptance. The second situation type is named *care potential,* which places the user in the latitude of noncommitment, where the suggestion to seek care still has potential. These situations are defined by doubt about seeking human care and either a negative or severe situation, or a negative stance and a severe situation. The final situation type we will call *care rejected*, which is defined by a non-severe situation and doubt about seeking care, or a negative stance towards seeking care and a medium or low severity situation. These place the user in the latitude of rejection, assuming that the suggestion to seek human care will be not be successful. The model proposes a different strategy for each of the situation types.

**Fig. 4:**
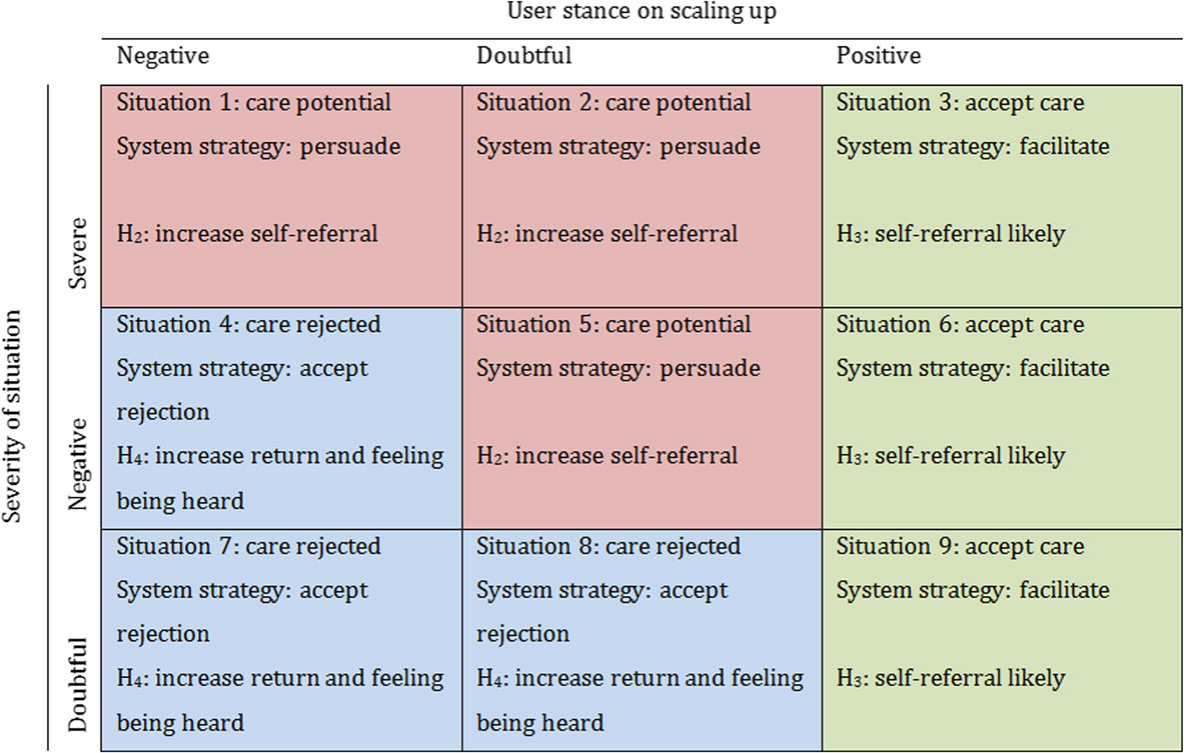
Situations set by health severity and user stance on scaling up

Figure 4 presents a systematic description of the nine possible situations as shown in Fig. 3, as well as the proposed referral strategy for every situation. The situations can be divided into three groups. The first are the *accept care* situations in which the user is likely to self-refer. The second group are the *care potential* situations in which the user is less likely to self-refer, but there is still potential. The final group are the *reject care* situations, in which the user is likely to reject the suggestion to seek care. For each situation group, the model proposes a different referral strategy.

When users are in the *accept care* situation, model 3 proposes the *facilitate* strategy. This strategy is based on two different theories of behaviour change. Fogg (2009) describes a theory where the combination of motivation and ability need to be high enough for a trigger or que to do the behaviour to have effect [34]. Similarly, Michie et al. (2011) describe the behaviour change wheel that states that motivation, capability and opportunity all play a role [35]. To change behaviour, a strategy needs to change one of those three factors to be successful. In this *accept care* situation the motivation to self-refer is high. The model will therefore increase the capability of the user to do so by facilitating contact to a human caregiver, for instance by providing contact details. This facilitation also serves as trigger to provide the user with the opportunity to self-refer.

In the *care potential* situation a trigger is less likely to directly succeed, as motivation is lower. The protocol therefore proposes the *persuade* strategy in an attempt to increase the user’s motivation. After this, it will announce the facilitation of contact. If the user does not actively object, the model will automatically continue with triggering the user by providing opportunity to self-refer. This means that the default option is to facilitate, although the user might still stop the system if they were not convinced by the motivation. This implementation of a default strategy is done because people have a tendency to go with a default option [36].

For users in the *reject care* situation a different approach is taken. Because users are in the latitude of rejection, the suggestion of self-referral might have an adverse effect. Therefore, the strategy is to *explicitly accept* that the user rejects human care, but to still give users the opportunity to contact a human in case they change their mind. In this way, the model aims at increasing the user’s intention to return to the system in the future, and how much the user feels the system really listened to them. It should be noted that the situation with a negative stance on self-referral and a severe situation (situation 1 as defined by Fig. 4) is not a *reject care* situation despite the low stance on scaling up. The reason for this is the severity, meaning that there is little to lose by trying to persuade the user despite the low chance of that succeeding.

Figure 4 shows the different situations as defined by stance on scaling up and situation severity, along with the situation type, the matching referral strategies and underlying hypotheses. These four hypotheses of the motivation model are as follows:

- *H*_*1*_: Agent personalisation in terms of its communication strategy, based on the situations characterised by user stance on seeking care and the severity of health risk situation, has an effect on the user’s intention to self-refer, their likeliness of contacting the agent again, and their feeling of being heard by the agent.
- *H*_*2*_: In situations with care potential, users are more inclined to self-refer if the agent provides persuasive messages instead of given no messages or providing only referral information.
- *H*_*3*_: In situations where users are likely to accept health care, they are inclined to self-refer if the agent facilitates referral.
- *H*_*4*_: In situations where users are likely to reject advise to seek health care, users are more likely to contact the agent again, and feel that the agent has heard them better if the agent accepts this rejection instead of persuading to self-refer or only facilitating referral.

### Experimental method

Model 3 is particularly suited for empirical evaluation, given the four hypotheses that underlie it. Both the detection and the auto-referral models are less suitable for empirical evaluation, however, as their details need to be specified for different applications. These models provide a framework for the implementation of procedures and technology. Whether these procedures are suitable for a specific AEMH system would have to be determined per intervention. Therefore, this section describes an experiment performed to study the hypotheses on the motivation model as presented in the previous section.

An experiment with a 3 × 3 × 3 design was performed, studying the effect of situation severity, initial stance on seeking human care and agent strategy on intention to self-refer, intention to return to the agent and the feeling of being heard by the agent. Hypothesis 1 was tested over all data, while hypotheses 2, 3 and 4 were tested on subsets of the data representing their respective relevant situations. Participants were recruited online from the general population and were asked to imagine they were having problems following scenarios representing the nine possible situation types. The domain chosen for this experiment was that of sleeping problems as many people have had at least some measure of experience with sleeping problems, and about a third meet at least one criteria for clinical insomnia in their lifetime [37]. The AEMH system used for this experiment was a virtual agent with chat function.

#### Participants

Two hundred twenty nine participants were recruited via Amazon’s Mechanical Turk and paid 1$ for their time. Participants were excluded if they had not read the consent form properly, if they could not confirm having seen the virtual agent, if they did not complete the survey, or if they did not complete the chats with the virtual agent. 160 participants were eventually included in the study, all were native English speakers. Age, gender and insomnia scores for all participants can be found in Table 1.

**Table 1:** Demographic characteristics of participants (and deviation of neutral score)

Characteristics		*F*(1,318)	*p.* value
Age in years, mean (SD)	36 (10.41)		
Gender, n (%)			
Female	91 (56.88)		
Male	67 (41.88)		
Other	2 (1.25)		
Insomnia severity index score, Mean (SD)	17 (6.53)		
No insomnia, n (%)	10 (6.31)		
Sub-threshold, n (%)	54 (33.46)		
Moderate severity insomnia, n (%)	55 (34,57)		
Severe insomnia, n (%)	41 (25,65)		
Main concern not seeking care			
Money, n (%)	128 (79.92)		
No problem, Mean in $ (SD)	24.76 (20.66)		
Maybe a problem, Mean in $ (SD)	39.68(22.27)		
Problematic, Mean in $ (SD)	57.01(27.59)		
Travel time, n (%)	15 (9.29)		
No problem, Mean in hours (SD)	0.67 (0.47)		
Maybe a problem, Mean in hours (SD)	1(0.58)		
Problematic, Mean in hours (SD)	1.85(1.07)		
Social stigma, n (%)	17 (10.78)		
Family, n (%)	5 (29.41)		
Friends, n (%)	3 (17.65)		
Boss, n (%)	12 (70.59)		
Intention to self-refer, mean (SD)	0.76 (1.85)	52.28	<.001
Intention to contact agent again, mean (SD)	−0.03 (1.98)	0.05	.824
Feeling of being heard by agent, mean (SD)	0.79 (1.66)	52.09	<.001

For the main personal concern not to seek human care, also the values entered for ‘no problem’, ‘maybe a problem’ and ‘definitely a problem’ (money per hour, travel time in hours), or social group most likely to be a problem (social stigma)

#### Measures

#### Primary outcomes

*Intention to self-refer (ISR):* Participants were asked to indicate how likely they would be to self-refer after the conversation with the agent, given their imagined situation. This question was answered on a 7-point scale ranging from *Extremely unlikely* via *Neutral* to *Extremely likely*.

*Intention to contacting agent again (ICAA):* The second primary outcome measure was studied with the question how likely the participant would be to contact the virtual agent again if they would have sleeping problems in the future. This question was answered on a 7-point scale ranging from *Extremely unlikely* via *Neutral* to *Extremely likely*.

*Feeling being heard (FBH):* Finally, a measure was in place to study how much participants felt the virtual agent heard them and took them seriously. This was measured with a seven-item questionnaire, all questions answered on a 7-point scale ranging from *not at all* via *neutral* to *very much*. This questionnaire contained two questions from the patient satisfaction questionnaire (PSQ) [38] (number 1 and 4), two from the trust in physician scale [39] (number 5 and 8) and three additional questions. Questions from the PSQ and trust in physician scale were slightly adapted to apply to virtual agents instead of doctors, were phrased as statements and negative statements were phrased positively to avoid double negatives in the scale answers. Reliability for this scale was measured for all 9 situations as participants filled in the scale three times for different situations. Cronbach’s alpha was between ɑ = 0.93 and ɑ = 0.97, showing high reliability.

##### Descriptive measures

Two descriptive measures were in place. Firstly, the insomnia severity index (ISI) was presented to all participants at the start of the experiment to check if experience with insomnia made a difference in the behaviour of the participants. Secondly, all participants were asked what their main reason would be to not seek human care. The options were money, travel time and social stigma. These options were later used to manipulate the situations, so if the answer was money the situation would refer to the potential cost of therapy.

##### Manipulation check

During the interaction, the virtual agent asked the participant how severe they would rate their situation, and how they felt about seeking human care based on the scenario they were given. These questions were in place to check if the participant’s interpretation of the scenario was as intended. They were implemented as multiple-choice options with three options so the answers could be compared to the three-tiered design of the scenarios.

#### Virtual agent chat

**Fig. 5:**
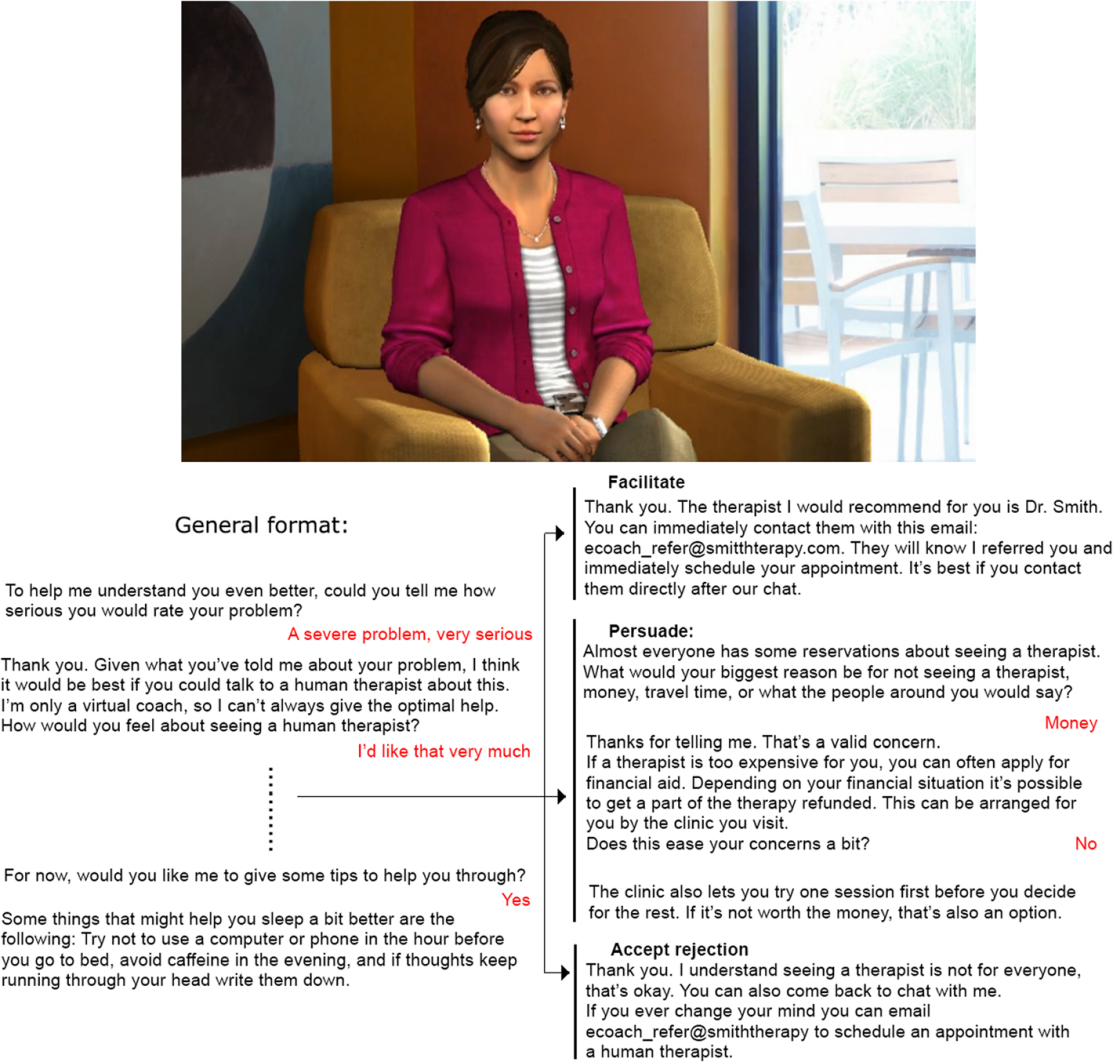
Virtual agent and the different referral strategies (differences shown on the right). Please note that small variations are possible. E.g. if people answer ‘no’ to the tips, these would not be given

The virtual agent chat was realized with the Roundtable Authoring tool developed by the USC Institute for Creative Technologies. The chats included questions asking after the severity of the situation and initial motivation of the participant to self-refer. A female virtual agent was displayed on the left of the screen while the chat was displayed on the right. Figure 5 shows the virtual agent and examples of the text for all three referral strategies.

#### Procedure

Because it was not known beforehand if participants suffered from sleeping problems, let alone how severe these were, or how people would feel about seeing a human therapist, participants were asked to imagine they were in a given scenario. These scenarios described a situation with regards to the severity of the sleeping problem and how likely the person was initially to self-refer. This second factor was personalized to the participant. At the start of the experiment, participants were asked if time, money or stigma would be the most likely factor to stop them from seeking help from a therapist. For those answering time or money, the next question would be how much time/money would be no problem, perhaps a problem or definitely a problem. In the case of stigma, participants were asked if telling their friends, family or boss would be the biggest problem. In the scenarios, the answers to these questions were used to describe a situation in which seeing a human therapist would be no problem, perhaps a problem or definitely a problem. For example; if the participant’s answers were *money* and *50 dollars would definitely be a problem*, the scenario describing a negative stance towards seeing a therapist would state that the only possible clinic would cost at least 50 dollars.

Each participant was randomly presented with three of the nine possible scenarios describing a level of insomnia severity and initial stance towards self-referral (See Additional file 1: Appendix 1 for an example of the scenario). For each scenario, participants were first asked to read the text and imagine they were in this situation. Afterwards, they were redirected to a link where they could chat with a virtual agent. The virtual agent would either *accept that they rejected care*, try to *persuade* them to seek help, or immediately *facilitate referral* to human care. During the whole experiment, all possible 9 (3 motivation × 3 severity) situations were mixed with all three referral strategies. Which three of the total of 21 possible combinations a participant saw and in what order was randomized. After the chat, participants were asked a number of questions about how likely they would be to self-refer after the conversation, how likely they would be to return to the agent and if they felt the agent really heard them.

#### Data preparation & analysis

Data was analysed with R version 3.3. For both the feeling of being heard scale and the ISI scale scores to the questions were averaged to create a single score. For the FBH scale, the ICAA and ISR the answers were transformed into a score between − 3.5 and 3.5 so deviation from the neutral 0 could be calculated. The full dataset is available online [40].

##### Descriptive outcomes

The primary outcome measures were examined to study whether users generally intended to self-refer after the chat, to return to the agent and if they felt being heard. Multilevel analyses were done taking participant as random intercept to control for the multiple measures per participant. Null models were run to reveal the deviance from the neutral point of zero for all dependent variables, while situation severity and motivation to seek help were each added as fixed effect to reveal if they further affected the dependent variables.

##### Manipulation check

Every participant imagined a total of three out of nine possible situations during the experiment, varying in the severity of their problem and the initial motivation to self-refer. To ensure that these scenarios were interpreted correctly by the participants, they were also asked by the virtual agent how they would rate these two situational variables. Cumulative linked mixed models with participant as random intercept were run to study if the situations as described in the scenarios correctly predicted how the situation was interpreted.

##### Covariate check

Covariate checks were performed for participant age, gender, ISI scores and the reason why participants would not seek help from a human therapist. These reveal that only gender had an effect, namely on FBH score (*F*(2, 156) = 4.01 *p* = .02), male participants giving higher scores (Male *M* = 1.15 *SD* = 1.53, Female *M* = 0.54 SD = 1.70, Other *M* = 0.29 *SD* = 1.38).

##### Hypotheses

Multilevel analyses were conducted for each of the nine situations, including only a subset of two agent strategies (e.g. persuasion and reference only) on the three outcome measures: ISR, ICAA, and FBH. Null models included participant as random intercept and for FBH gender was included as fixed factor. The extended model also included agent strategy as fixed factor. The null models and extended models were compared to test the effect of agent strategy.

The first hypothesis was tested using a multilevel analysis on ISR, ICAA and FBH scores. This null model only included a fixed intercept and participant as random intercept. Model 1 built on the null model and added situation as fixed effect. Model 2 also built on the null model but added agent strategy instead. Model 3 built on model 2 and added situation as fixed effect as well. Finally model 4 was the full model that built on model 3 and included two-way interaction between situation and agent strategies as fixed effect. Comparing these models tested the added contribution of fixed effects.

The second hypothesis was tested with a multilevel analysis on intention to self-refer data by only including the subset of situations with care potential, i.e. situations 1, 2 and 5. The null model included participant as random intercept and fixed intercept. Model 1 built on model 0 and added agent strategy. After comparing model 0 and 1, the analysis was repeated on two subsets: one that include only persuasion and no action as agent strategy, and the other subset that included only persuasion and facilitate only as agent strategy.

The third hypothesis was tested with a single multilevel analysis on data only including situations 3, 6 and 9, in which the agent followed the facilitate strategy. This analysis only included a null model on intention to self-refer that included fixed intercept effect and participants as random intercept effects. The hypothesis was examined by considering whether fixed intercept significant deviated from zero.

The fourth and final hypotheses were tested with two multilevel analyses, for both how likely participants are to contact the agent again, and how much they felt being heard by the agent. A null model was run including fixed intercept effect and participants as random intercept. The hypothesis was examined by comparing the null models to a model also including agent strategy. Furthermore, null models and models including agent strategy were also compared in two subsets: one that include only persuasion and no action as agent strategy, and the other subset that included only no action and facilitate only as agent strategy.

## Result

### Descriptive outcomes

Table 1. shows the demographic characteristics of participants. This table shows that the main reason given for not seeking care was money. Average insomnia scores were high, with one-fourth of participants meeting the criteria for severe insomnia and one-thirds for moderate severity insomnia. For the dependent variables, ISR and FBH were both significantly above neutral, participants indicating that they would self-refer in this situation, and that they felt being heard by the agent. ICAA scores do not show this pattern.

### Manipulation check

To confirm if the participants interpreted the scenarios about sleep as they were intended, a manipulation check was performed. This analysis shows that scenario description of the situation significantly predicted the subjective interpretation for both situation severity (χ^2^_1_ = 142.98, *p* < .0001) and initial motivation to self-refer (χ^2^_1_ = 184.49, *p* < .0001). Pseudo R^2^ was .14 for severity and .18 for motivation as calculated following [41], effect size was calculated using the χ^2^test for goodness of fit was large for both severity at w = .55 and motivation at w = .62 [42]. This indicates that largely, situations as presented in the scenarios were interpreted correctly by the participants.

### Hypothesis 1: Personalisation agent communication strategies

**Fig. 6:**
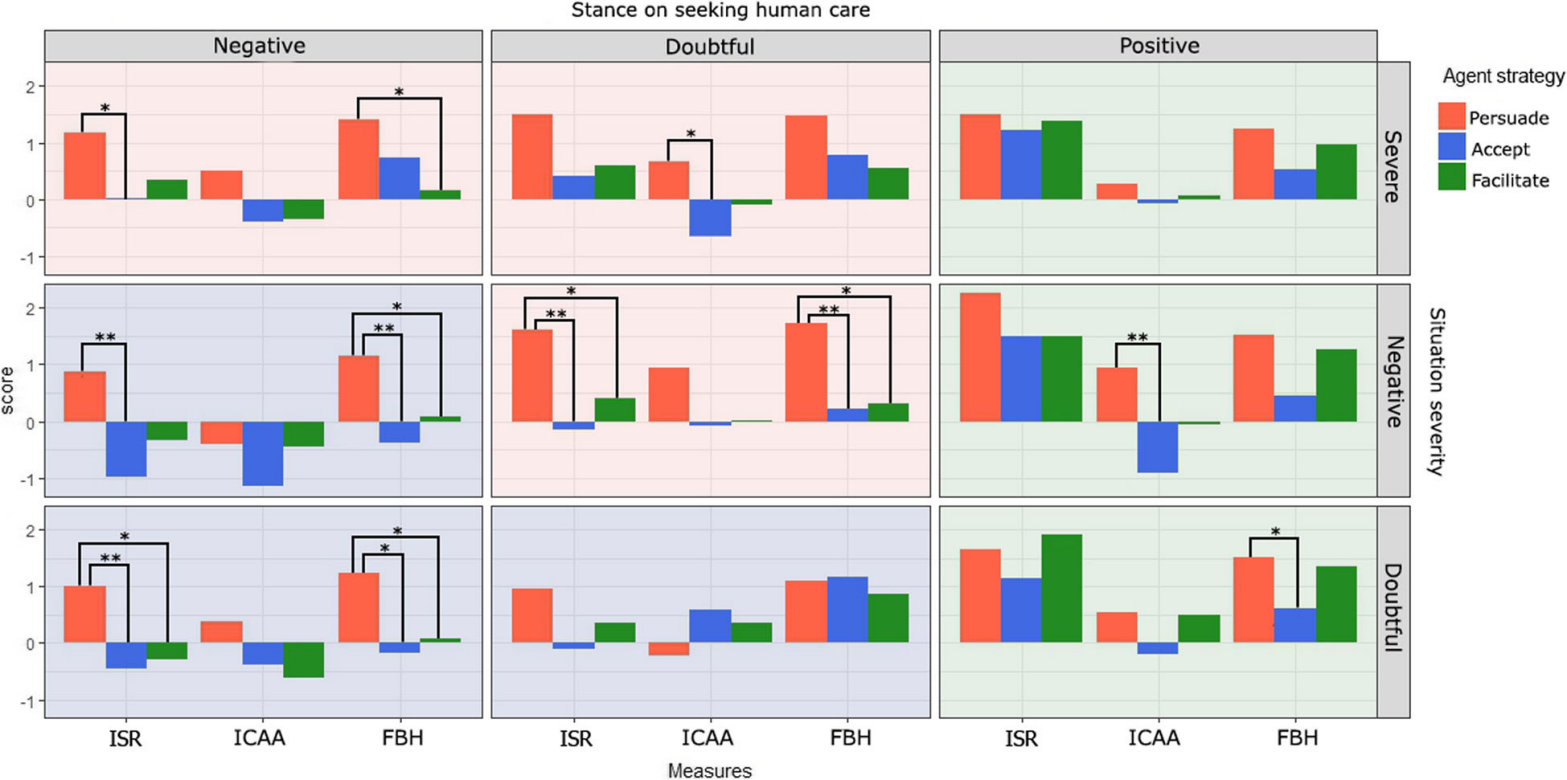
Comparison of agent strategies over the 9 different situations. * Represents *p* < 0.05, ** *p* < 0.01

Table 2 shows the results from the analysis for hypothesis 1. This table shows that both situation and agent strategy significantly influenced intention to self-refer, intention to contact the agent again, and the feeling of being heard. The interaction between situation and agent strategy was not found to affect any of these measures. To further study interaction, this test was repeated on datasets excluding one of the agent strategies, revealing that for *accept rejection* and *facilitate only*, an interaction was present for feeling of being heard (χ^2^_8_ = 18.46, *p* < .018), as for *persuade* and *facilitate only* (χ^2^_8_ = 18.91, *p* < .015). Examining Fig. 6. it seems that especially for the *facilitate only* strategy, the interaction between agent strategy and situation influences how much participants feel being heard by the system, this strategy working better when initial stance on self-referral is higher.

**Table 2:** Multilevel analysis results on seeking care on all situations: model comparisons

Model comparison	*n*	χ^2^	*p*
*Intent to self-refer*			
Situation (M_0_ vs M_1_)	477	χ^2^_8_ = 69.84	<.001
Agent strategies (M_0_ vs M_2_)	477	χ^2^_2_ = 33.80	<.001
Situation × Agent strategies (M_3_ vs M_4_)	477	χ^2^_16_ = 13.67	0.623
*Intent to contact the agent again*			
Situation (M_0_ vs M_1_)	477	χ^2^_8_ = 18.30	0.019
Agent strategies (M_0_ vs M_2_)	477	χ^2^_2_ = 28.26	<.001
Situation × Agent strategies (M_3_ vs M_4_)	477	χ^2^_16_ = 11.80	0.758
*Feeling of being heard*			
Situation (M_0_ vs M_1_)	477	χ^2^_8_ = 17.51	0.025
Agent strategies (M_0_ vs M_2_)	477	χ^2^_2_ = 47.02	<.001
Situation × Agent strategies (M_3_ vs M_4_)	477	χ^2^_16_ = 19.18	0.260

### Hypothesis 2: Persuasion in situations with care potential

Table 3 shows that in situations with care potential, the agent strategy significantly effects intention to self-refer. Further analysis shows that the persuasion strategy significantly differs from the other two strategies, Fig. 6 showing that persuasion leads to higher scores.

**Table 3:** Multilevel analysis results on seeking care in care potential situations (1, 2, and 5): model comparisons

Model comparison	*n*	χ^2^	*p*
Agent strategies (M_0_ vs M_1_)	173	χ^2^_2_ = 17.83	<.001
Sub set agent strategies			
Persuasion vs Accept rejection (M_0_ vs M_1_)	111	χ^2^_1_ = 12.40	<.001
Persuasion vs Facilitate (M_0_ vs M_1_)	116	χ^2^_1_ = 11.29	<.001

### Hypothesis 3: Providing referral information in situation where user likely to accept health care

In situations 3, 6 and 9 as described in Fig. 4, intention to self-refer after interaction with the virtual agent that facilitated contact significantly deviated from the neutral value of zero (*F*(1,147) = 59.04, *p* < .0001). Figure 6. shows that participants would seek care in the *accept care* situation after the agent used the *facilitate* strategy.

### Hypothesis 4: No messages in situation where users reject advise to seek health care

Table 4 shows the results from the analysis for hypothesis 4. In the *reject care* situation, agent strategy did not affect the participant’s intention to contact the agent again. Strategy did have an effect on the feeling of being heard, Fig. 6 shows that here the persuasion strategy outperformed the accept rejection strategy.

**Table 4:** Multilevel analysis results on intent to contacting the agent again, and feeling agent has heard them in situations that user reject advice to seek health care (4, 7, and 8): model comparisons

Model comparison	*n*	*χ* ^*2*^	*p*
*Intent to contact the agent again*			
Agent strategies (M_0_ vs M_1_)	152	χ^2^_2_ = 1.00	.605
Sub set agent strategies			
Accept rejection vs Persuasion (M_0_ vs M_1_)	99	χ^2^_1_ = 0.59	.441
Accept rejection vs Facilitate (M_0_ vs M_1_)	102	χ^2^_1_ = 0.03	.874
*Feeling agent has heard them*			
Agent strategy (M_0_ vs M_1_)	152	χ^2^_2_ = 16.60	<.001
Sub set agent strategy			
Accept rejection vs Persuasion (M_0_ vs M_1_)	99	χ^2^_1_ = 12.03	<.001
Accept rejection vs Facilitate (M_0_ vs M_1_)	102	χ^2^_1_ = 0.003	.953

### Discussion experimental results

The demographic results show that more than half of the participants met the criteria for clinical insomnia. These high numbers could be partly caused by a potential response bias, specifically the Hawthorne effect, where participants behave differently because they are in an experimental setting. The Insomnia Severity Index is usually only applied after sleeping problems have already been reported and the questions may have guided some participants to report more severe symptoms. Even taking a conservative stance and expecting that severity of insomnia problems to be lower, it seems likely that some level of insomnia was existing in this sample, which suggests that the sleep domain was fitting for this participant group. Score on the ISI did not affect the outcome measures in the experiment, which indicates that having sleeping problems does not affect the interpretation of a scenario about sleeping problems.

Overall, both situation (stance on seeking human care and severity) and agent strategy (facilitate, persuade or accept care) affected intention to self-refer, intention to return to the agent and the feeling of being heard. However, results show no interaction effect between situation and the three referral strategies used by the agent. Still, an interaction effect was found on *Feeling of Being Heard* as long as the *facilitate* strategy was included when comparing two strategies at a time. This shows that for the *facilitate only* strategy especially, the *feeling of being heard* was influenced by the interaction between situation and strategy. This could indicate that situation does matter for the effect of the *facilitate* strategy, but less for the other two. The results therefore only partly support hypothesis 1. When considering the individual situation types, results show that users in the *care potential* situations are more likely to seek care when the agent uses the *persuade* strategy than if it uses *accept rejection* or *facilitate* strategy. So, when participants are in doubt whether they wish human care, persuading them to self-refer is effective, confirming hypothesis 2. In the *accept care* situations, results show that participants are inclined to self-refer after the *facilitate* strategy. This supports hypothesis 3 and indicates that if a user is motivated to seek care, simply providing information is enough to get them to do so.

The final situations are the *reject care* situations, in which initially motivation to self-refer is low. The focus of the model therefore lies on increasing the intent to contact the agent again and the feeling of being heard through the *accept rejection* strategy. Results show, however, that the *persuade* strategy has a better effect on the feeling of being heard, while no difference between strategies is found for the intention of contacting the agent again, thus not confirming, nor giving grounds for rejecting, hypothesis 4. Hypothesis 4 was based on the assumption that if initial motivation to seek care is low the user is in the latitude of rejection for the suggestion to self-refer. That the results do not reflect this assumption might have two reasons. The first would be that initial motivation to seek care was indeed low, but the latitude of rejection simply does not exist for the suggestion to self-refer. If this is indeed the case, the model would need to be adapted to reflect this. Another possibility is that the manner in which an initial low motivation was manipulated in this experiment was not actually strong enough to achieve the latitude of rejection. Motivation was framed in terms of money, time and how many people would be told of therapy. All of these concerns could be partially taken away by the agent in the *persuasion* strategy. However, in real-live situations, low motivation to seek care might be more difficult to dispel. The trans-theoretical model of behaviour change [43] identifies the *precontemplation stage* where people might not even agree their situation warrants action, in which case it would be more difficult to persuade people to self-refer. Also, not all reasons to not self-refer could be taken away as easily as those used in the scenarios in this experiment. This could mean that the simulated initial low motivation situation in the experiment did not accurately reflect a real-life low motivation.

## Discussion

In this paper three protocols are presented, which together cover how to detect risk situations in AEMH applications and how to act once those risks are detected. Based on existing models of risk detection, theoretical models from behaviour change and expert interviews, these proposed models provide a base for designing risk detection in AEMH systems. The final model, on how to motivate users to seek human care, was further empirically evaluated. This evaluation revealed that how referral strategy is personalised to different user situations influences intention to self-refer, intention to visit the agent again and the feeling of being heard.

In the design of the protocols, care was taken to ensure they would be generalizable to a broad range of different AEMH systems. This means that they do not include specific questionnaires, thresholds, algorithms or information about user groups which might not be applicable to all systems. So, they could be used by systems that differ in target group, goal, platform and technical capabilities. However, the protocols do not fill in all the details, as those would need to be specified for every system separately. Given the broad range of AEMH systems in use [44, 45] this generalizability is important for any risk framework.

The protocols described in this paper are meant to be applied to AEMH systems, but are also relevant for conventional care. When an AEMH application detects a risk it cannot deal with, human care becomes important again. This fits in with the concept of blended, or stepped care, in which technological and traditional approaches are combined to provide people with the best care possible [5]. Application of the auto-referral protocol in particular, should be done in consultation with the health-care professionals involved. Similarly, the protocols also highlight the importance of *concordance,* where the patient’s opinions and thoughts are a crucial part of the decision-making process on their health-care [46]. Although initially used to aid discussions about medication adherence, this concept could also be applied to situations in which patients and providers may need to reach mutual decisions about health interventions, including the choice to refer as described in the motivation model.

To fully appreciate the protocols presented in this paper some limitations should be considered. The detection model and auto-referral model were not empirically evaluated in this paper. These need to be applied for a specific system and intervention, and experts would need to evaluate their fit for different purposes. However, by basing these models on literature, existing models, and specific input from experts, the protocols form a solid base to designing risk management. In the evaluation of the *motivation model* participants were recruited via crowd-sourcing. This has the limitation that participants belong to a demographic used to working with online services. However, an increasing number of AEMH systems are deployed online as well. Another limitation to this form of recruitment is that participants did not necessarily have sleeping problems. Instead, they were asked to imagine their situation and, after chatting with a virtual agent, indicate what their actions would be on a subjective scale. Although a more feasible methodology than recruiting participants with confirmed sleeping problems and studying actual behaviour, the possible effects of having imaginary situations and measuring subjective intention should be taken into account. The results from this study confirmed some of the hypotheses formulated in section 2.2.2, but not all. The *reject care* scenarios in particular, were meant to describe a situation in which the suggestion to self-refer would not be accepted. However, from the results we see that this suggestion was still effective in some cases, indicating that it did not fall into the latitude of rejection after all. Further research is necessary to study if this latitude of rejection does exist for this type of scenario, and what referral strategy would be most appropriate.

Several other directions for future research can be established. Applying the detection model in different types of AEMH systems could reveal whether any gaps exist in the model. If any decisions in risk detection for a specific system are not covered by the model, this knowledge could serve to improve the model. Furthermore, application of these models also opens up the possibility of obtaining data which may be used to test and refine the protocols. This model could also be extended with more specific instructions for particular risk situations. The first situation to cover would be suicide detection, as this is still a critical problem within mental health care [47, 48]. These suggestions could take the form of what keywords to detect, what questionnaires to use, what threshold values to use, etc. Similarly, the auto-referral model could be evaluated after being applied into specific systems. This model currently operates on the premise that the user is aware they can be automatically referred. However, some risks might be so severe that safety takes priority over consent. Exactly how this would change the model is another avenue for further study. Finally, as technology advances it would also be important to re-evaluate the models to ensure that they stay applicable for new systems.

## Conclusion

This paper shows the possibility of formal and generalizable models for risk detection in AEMH systems. These models represent a first step towards risk detection and management in e-mental health care. The detection model shows two important steps; namely detecting a possible risk situation and deciding if this situation is severe enough to seek human care. The second model considers auto-referral and stresses the importance of expectation management and the role the user can still play in seeking contact. The third model considers motivating users to seek care themselves. An empirical evaluation shows that the strategy employed by the system influences users’ intention to self-refer, intention to return to the system and the feeling of being heard. Given the inability of conventional health systems to manage the demand for mental health services, AEMH systems are set to become more common, but these must be both cost-effective and safe. The models outlined here provide a valuable foundation on which to build risk detection and management into these systems.

## Additional file

Additional file 1: **Appendix 1.** Examples scenarios. This appendix shows examples of the different scenarios provided to the participants to convey their imagined situation. (DOCX 13 kb)

## Abbreviations

AEMH: Autonomous E-Mental Health
FBH: Feeling being heard
ICAA: Intention to contacting agent again
ISR: Intention to self-refer
PSQ: patient satisfaction questionnaire
PTSD: Post-traumatic stress disorder
WHO: World Health Organisation
