# Supplementary material for: Considering patient safety in autonomous e-mental health systems – detecting risk situations and referring patients back to human care
Source: BMC Med Inform Decis Mak. 2019 Mar 18;19:47. doi: 10.1186/s12911-019-0796-x (PMC6421702; doi:10.1186/s12911-019-0796-x)
Supplement: Supplementary file 1 — Appendix 1. Examples scenarios. This appendix shows examples of the different scenarios provided to the participants to convey their imagined situation. (DOCX 13 kb) [file 12911_2019_796_MOESM1_ESM.docx]

**Additional file 1: Appendix I – Examples scenarios**

**Low motivation, High severity situation (Money)**

You’ve been having problems sleeping. You keep waking up at night, staying awake for hours at a time. You’ve been very tired and not functioning well at all. It’s been going on for a couple of months now, and sometimes you’re awake nearly the whole night. Lately, it seems to have been getting worse.

You've doing some research on getting help for this problem. There's only one possible clinic you could go to for this problem. Seeing the therapist will cost you XXX dollar per hour. This is not an option for you, so you've come to the virtual coach to see if it can help you instead.

**Medium motivation, medium severity (Stigma)**

The last couple of weeks you’ve been having some sleeping problems. You’re sleeping very fitfully, waking up a little and dreaming a lot. In the morning, you feel barely rested. It’s starting to affect how you’re functioning. It’s been going on for a couple of weeks now.

You've doing some research on getting help for this problem. There's only one possible clinic you could go to for this problem. You’d have to tell your XXX that you have an appointment, but you can avoid them it’s with a therapist. That's only just doable for you, so you've been having doubts. Instead, you've decided to chat with the virtual coach to see if it can help you instead.

**High motivation, low severity situation (Time)**

You’ve been having some sleeping problems. You used to fall asleep immediately, but lately it’s been taking a bit longer, up to an hour. You’re still getting enough sleep functioning okay, but it’s annoying. It has been going on for a couple of weeks now.

You've doing some research on getting help for this problem. There's only one possible clinic you could go to for this problem. It will take you XXX hours to get to the clinic to see the therapist. That's very doable for you, so you'd like to go there. You couldn't find their contact info, however, so you've decided to chat with the virtual coach first to see if it can help you.
